# Validity and reliability of the Neilands sexual stigma scale among Kenyan gay, bisexual, and other men who have sex with men

**DOI:** 10.1186/s12889-022-13066-3

**Published:** 2022-04-14

**Authors:** Christine J. Korhonen, Brian P. Flaherty, Elizabeth Wahome, Pascal Macharia, Helgar Musyoki, Parinita Battacharjee, Joshua Kimani, Monika Doshi, John Mathenge, Robert R. Lorway, Eduard J. Sanders, Susan M. Graham

**Affiliations:** 1 Department of Epidemiology, University of Washington, Box 357236, Seattle, USA; 2 Department of Psychology, University of Washington, Box 351525, Seattle, WA 98195 USA; 3 Kenya Medical Research Institute-Wellcome Trust Research Programme, Kilifi, Kenya; 4 Health Options for Young Men on HIV/AIDS and STIs, Nairobi, Kenya; 5 National AIDS and STI Control Programme, Ministry of Health, Nairobi, Kenya; 6 Partners for Health and Development in Africa, Nairobi, Kenya; 7 University of Manitoba, Winnipeg, Canada; 8 Saath, Connecticut, USA; 9 University of Oxford, Oxford, UK

**Keywords:** Social stigma, Homophobia, Surveys and questionnaires, Sexual and gender minorities, Factor analysis

## Abstract

**Background:**

We evaluated the validity and reliability of the Neilands sexual stigma scale administered to 871 gay, bisexual, and other men who have sex with men (GBMSM) at two research locations in Kenya.

**Methods:**

Using cross-validation, exploratory factor analysis (EFA) was performed on a randomly selected subset of participants and validated using confirmatory factor analysis (CFA) on the remaining participants. Associations of the initial and final stigma scale factors with depressive symptoms, alcohol use, and other substance use were examined for the entire dataset.

**Results:**

EFA produced a two-factor scale of perceived and enacted stigma. The CFA model fit to the two-factor scale was improved after removing three cross-loaded items and adding correlated errors (chi-squared = 26.5, df 17, *p* = 0.07). Perceived stigma was associated with depressive symptoms (beta = 0.34, 95% CI 0.24, 0.45), alcohol use (beta = 0.14, 95% CI 0.03, 0.25) and other substance use (beta = 0.19, 95% CI 0.07, 0.31), while enacted stigma was associated with alcohol use (beta = 0.17, 95% CI 0.06, 0.27).

**Conclusions:**

Our findings suggest enacted and perceived sexual stigma are distinct yet closely related constructs among GBMSM in Kenya and are associated with poor mental health and substance use.

## Introduction

Gay, bisexual, and other men who have sex with men (GBMSM) experience stigma and discrimination in Kenya and other countries, especially where same-sex sexual behavior is criminalized [1, 2]. Chronic stress from experiences of sexual stigma contributes to mental and physical health disparities among GBMSM, and can be compounded, for some individuals, by intersecting stigmas such as those surrounding living with HIV or engaging in transactional sex [3–11]. While the World Health Organization (WHO) and Joint United Nations Progamme on HIV/AIDS (UNAIDS) recommend stigma reduction and anti-discrimination measures as part of a comprehensive HIV and STI prevention approach [12, 13], a better understanding of stigma surrounding same-sex sexual behavior in the Kenyan context is needed in order to develop effective, multi-pronged approaches.

Stigma is multi-dimensional and has been classified into perceived, enacted, and internalized forms [14]. Perceived stigma refers to expectations of negative opinions of one’s own group, along with fears of experiencing future discrimination. Enacted stigma includes direct encounters of violence or discrimination. Internalized stigma refers to the incorporation of discrediting social views into one’s own beliefs and opinions about oneself. Several instruments have been developed to assess each of these dimensions, such as Krieger’s Experiences of Discrimination and Mayfield’s Internalized Homonegativity Inventory [15, 16].

A 10-item scale evaluating perceived and enacted sexual stigma among men who have sex with men (referred to here as GBMSM to reflect the diversity of sexual orientation in this population [17]) was adapted by Neilands et al. [18] from a scale developed by Diaz et al. [19]. The Neilands sexual stigma scale has been used globally, including in South Africa and Kenya [20–22]. In coastal Kenya, higher Neilands sexual stigma scores were correlated with alcohol abuse, other substance abuse, and depressive symptoms [22].

The present study evaluated the reliability and validity of the Neilands sexual stigma scale adapted for use among GBMSM in Kenya. The specific aims were to describe the scale’s factorial structure and assess associations of this scale with self-reported levels of depressive symptoms, alcohol use, and substance use.

## Methods

### Study setting and participants

This secondary analysis used cross-sectional data from two research studies by members of the Kenya MSM Health Research Consortium, a collaboration focused on improving HIV prevention and care services for GBMSM in Kenya, and by Health Options for Young Men on HIV/AIDS and STIs (HOYMAS), the National AIDS and STI Control Programme (NASCOP), and Partners for Health and Development in Africa (PHDA).

Data from the Nairobi and coastal Kenya studies were pooled, as in previous research [23], to improve generalizability and to inform use of this scale in future research across multiple sites within Kenya. Mobility between locations is common, so care was taken to avoid duplicate enrollment.

Participants in both studies were at least 18 years of age. Researchers obtained written informed consent from all participants. This analysis was approved by the University of Washington Institutional Review Board.

#### Nairobi

Participants were recruited for a cross-sectional study on HIV vulnerability sponsored by the Canadian Institutes of Health Research, described previously [24]. This study grew out of a larger community-based research project, the South-to-South study, coordinated and supervised by HOYMAS community leaders with technical support from PHDA and the University of Manitoba. Recruitment took place at two health clinics providing services for GBMSM, one of which specifically targeted those who identified as sex workers, between January and May 2016. Men were eligible for participation if they were enrolled for services at either of the clinics and reported ever having had anal sex with a man. This study was the first evaluation of mental health and sexual stigma at these clinical sites using the measures described below.

#### Coastal Kenya

Participants were members of two ongoing cohort studies (one for HIV-positive and the other for HIV-negative adults at high risk for HIV transmission) based at the Kenya Medical Research Institute-Wellcome Trust Research Programme (KEMRI-WTRP) in Mtwapa [22, 25]. Recruitment was conducted at voluntary counseling and treatment centers adjacent to the research clinics or by peer recruiters at social venues such as night clubs, as described previously [26]. Sociodemographic data were collected at enrollment. Mental health data were collected at enrollment or at a follow-up visit from December 2015 through November 2017.

Men were eligible for the current analysis if they reported having had anal sex with a man in the past three months.

### Measures

#### Neilands sexual stigma scale

The Neilands sexual stigma scale consists of ten items (for example, “How often have you heard that homosexuals are not normal?” and “How often have you been hit or beaten up because you have sex with men?”) with response choices of ‘Never,’ ‘Once or twice,’ ‘A few times,’ or ‘Many times.’ Neilands’ exploratory factor analysis found two factors, perceived and enacted stigma, with one item (Item 3, “How often have you been made fun of or called names for being homosexual?”) loading on both factors. This item was dropped from Neilands’ final model [18].

#### Changes to Neilands sexual stigma scale

The Neilands sexual stigma scale was adapted for use in the KEMRI-WTRP cohorts in preparation for a planned trial of an adherence support intervention for GBMSM living with HIV [27]. Based on feedback from translators and study staff, the tag ‘for being homosexual’ was changed to ‘because you have sex with men’ on each item since many participants did not identify as gay or homosexual. An additional item, ‘How often have you experienced police harassment because you have sex with men?’ was added due to criminalization of same-sex sexual behavior in Kenya and was a priori expected to load on the enacted stigma factor. The Cronbach’s alpha for this scale was 0.85 in a previous study among the KEMRI-WTRP cohorts, indicating good internal consistency between items [22].

#### Additional measures

Depressive symptoms were assessed using the Patient Health Questionnaire 9 (PHQ-9) depression module, which has been validated for use in Kiswahili in Kenya [28]. Responses were revised from the PHQ-9 standard ‘Not at all,’ ‘Several days,’ ‘More than half the days,’ and ‘Nearly every day’ to ‘Not at all,’ ‘A few days,’ ‘Several days,’ and ‘Nearly all the days’ based on translator feedback. Summed responses from the nine items ranged from 0 to 27 [29]. Previous studies of the KEMRI-WTRP cohorts found Cronbach’s alpha of 0.86 for the PHQ-9, indicating good internal consistency [22, 23].

Alcohol use was assessed using the Alcohol Use Disorder Identification Test (AUDIT). Each statement was rated on a five-point scale ranging from ‘Never’ to ‘Daily or almost daily.’ Summed responses for the 10 items ranged from 0 to 40 [30]. Previous studies of the KEMRI-WTRP cohorts found Cronbach’s alphas ranging from 0.87 to 0.88 for the AUDIT, indicating good internal consistency [22, 23].

Other substance use was evaluated using the Drug Abuse Screening Test 10 (DAST-10). Participants either agreed or disagreed with each statement, and summed responses ranged from 0 to 10 [31]. Commonly reported substances used included *khat* (an addictive stimulant typically chewed), marijuana, and pain medications [23]. A Cronbach’s alpha of 0.78 was found in the KEMRI-WTRP cohorts for the DAST-10, indicating acceptable internal consistency [22].

### Translations

Questionnaires were translated from English to Kiswahili by two staff members, then back translated by two different staff members fluent in both languages. To ensure equivalence in meaning, a committee of researchers and translators held a harmonization meeting where discrepancies were resolved by consensus. At the Nairobi site, questionnaires were additionally translated and back translated by a community-based research team including GBMSM to ensure wording would be understood by study participants.

### Data collection

The sexual stigma items and other measures were asked at both the Nairobi and coastal Kenya locations using audio computer-assisted self-interview (ACASI) in English or Kiswahili. After completing the ACASI, participants debriefed with a counselor and were provided referrals for mental health services as needed.

### Data analysis

Descriptive statistics and Cronbach’s alphas were calculated for the summed Neilands sexual stigma scale score overall and separately for each site. Spearman’s rank-order correlation coefficients were calculated for correlations between the summed Neilands sexual stigma scores and the summed scores for the mental health and substance use variables (i.e., PHQ-9, AUDIT, DAST-10).

We divided participants into two groups using the random number generator, ensuring equal numbers from each site. For cross-validation, exploratory factor analysis (EFA) was performed on one subset of participants and validated using confirmatory factor analysis (CFA) on the remaining participants. Polychoric correlations were used as appropriate for ordinal Likert responses. We used oblique rotation for EFA and weighted least squares with adjustment for means and variances estimation for categorical variables (WLSMV) for CFA [32, 33].

EFA factors were assessed using scree plots and factor loadings. Following standard guidelines, we considered loadings of 0.40 or greater for to be significant. Items with significant loadings on multiple factors were considered cross-loaded. For differences between cross-loadings of 0.20 or more, we assigned the item to the factor where it loaded most strongly [34].

We used currently recommended model fit criteria to guide CFA model selection: root mean square error of approximation (lower is better), comparative-fit index (higher is better), standardized root mean squared residual (lower is better), and chi-square statistic (non-significance is better) [35]. We tested both a one-factor and a two-factor model using all 11 items. To determine if results would have differed by site, we also conducted CFAs for each site separately, using all 11 items. Finally, post-hoc alterations were made on the model using the CFA sample, in order to improve model fit and reduce misspecification, as unmodeled measurement error can produced unpredictably biased results [35, 36]. Fit statistics from all CFA models were compared.

Structural equation modeling was used to calculate associations between the factors in the final sexual stigma model and the summed scores for depressive symptoms, alcohol use, and other substance use using the complete dataset. We compared those associations to associations calculated using the initial 11-item model before post-hoc alterations were made.

Descriptive statistics were calculated using Stata version 14.2 (StatCorp, College Station, TX, USA), while factor analyses and associations were conducted using MPlus version 8.4 (Muthen & Muthen, Los Angeles, CA, USA).

## Results

From the two locations, 880 participants completed the questionnaire. Nine who did not fully answer the stigma items were excluded, for a total of 871 participants (550 participants from Nairobi and 321 from coastal Kenya). Descriptive statistics are shown in Table 1, which presents characteristics overall, for the EFA and CFA samples, and for each site. Participants’ ages ranged from 18 to 64, with an average age of 27. Sixty-three percent (*n* = 548) had completed secondary school or had some higher education. Among participants, 54% (*n* = 466) reported engaging in transactional sex, and 30% (*n* = 258) were HIV positive. Cronbach’s alpha assessing internal consistency of the summed Neilands sexual stigma score was 0.86 overall, 0.88 in Nairobi, and 0.85 in coastal Kenya. The summed sexual stigma scale was weakly correlated with the mental health and substance use measures (PHQ-9, r_s_ = 0.40; AUDIT, r_s_ = 0.27, and DAST r_s_ = 0.24).

**Table 1:** Descriptive statistics

	Total *N* = 871	EFA Sample *N* = 435	CFA Sample *N* = 436	Nairobi *N* = 550	Coastal Kenya *N* = 321
Site^a^					
Nairobi	550	275	275	550	
Coastal Kenya	321	160	161		321
Age (mean, range)	27.1, 18–64	27.0, 18–55	27.1, 18–64	27.5, 18–64	26.4, 18–49
Married	59 (7%)	26 (6%)	33 (8%)	47 (9%)	12 (3%)
Religion					
Muslim	119 (14%)	70 (16%)	49 (11%)	50 (9%)	69 (22%)
Catholic	225 (26%)	108 (25%)	117 (27%)	142 (26%)	83 (26%)
Protestant	397 (46%)	204 (47%)	193 (44%)	307 (56%)	90 (28%)
None	91 (10%)	41 (9%)	50 (12%)	18 (3%)	73 (23%)
Other	39 (4%)	12 (3%)	27 (6%)	33 (6%)	4 (1%)
Education					
Less than primary to some secondary	323 (37%)	161 (37%)	162 (37%)	142 (26%)	179 (56%)
Completed secondary	289 (33%)	154 (36%)	135 (31%)	182 (33%)	107 (33%)
Some or completed higher education	259 (30%)	119 (27%)	140 (32%)	224 (41%)	35 (11%)
Transactional Sex	466 (54%)	241 (55%)	225 (51%)	383 (75%)	83 (42%)
HIV positive	258 (30%)	141 (32%)	117 (27%)	226 (41%)	32 (11%)

^a^Sample distribution was restricted by site

### Exploratory factor analysis

**Fig. 1:**
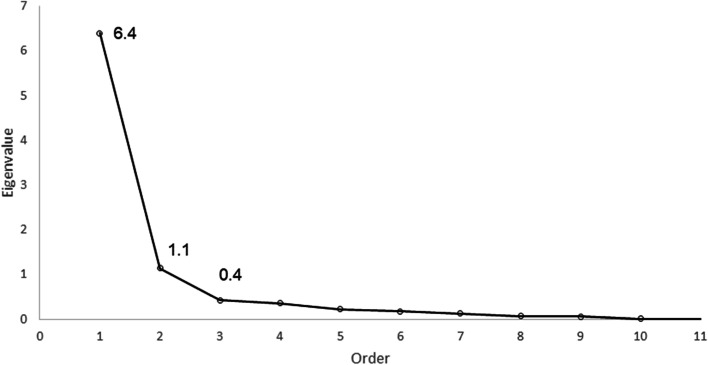
Exploratory factor analysis scree plot of the Neilands sexual stigma scale, *n*=435

The percent of respondents who reported experiencing each item are shown in Table 2. Among the 435 participants included in the EFA sample, the screeplot indicated a one- or two-factor solution, with an Eigenvalue of 1.1 for two factors (Fig. 1). Using two-factors, three items (1, 2, and 5) loaded on the perceived stigma factor and five items (4, 8, 9, 10, and 11), including the new item on police harassment, loaded on the enacted stigma factor (Table 3).

**Table 2:** Neilands sexual stigma scale items

Item	Question	Ever Experienced* N (%)
1	How often have you heard that homosexuals are not normal?	701 (80.5)
2	How often have you felt that you hurt and embarrassed your family because you have sex with men?	479 (55.0)
3	How often have you been made fun of or called names because you have sex with men?	507 (58.2)
4	How often have you been hit or beaten up because you have sex with men?	211 (24.2)
5	How often have you had to pretend that you are not homosexual in order to be accepted?	622 (71.4)
6	How often has your family not accepted you because you have sex with men?	274 (31.5)
7	How often have you lost your friends because you have sex with men?	453 (52.0)
8	How often have you been kicked out of school because you have sex with men?	130 (14.9)
9	How often have you lost a place to live because you have sex with men?	315 (36.2)
10	How often have you lost a job or career opportunity because you have sex with men?	245 (28.1)
11	How often have you experienced police harassment because you have sex with men?	269 (30.9)

*Response choices include: Once or twice, A few times, or Many times

**Table 3:** Factor loadings for the Neilands sexual stigma scale, *n* = 435

Item	Factor Loading	
	**Perceived Stigma**	**Enacted Stigma**
1.How often have you heard that homosexuals are not normal?	0.64	0.17
2.How often have you felt that you hurt and embarrassed your family because you have sex with men?	0.67	0.38
3.How often have you been made fun of or called names because you have sex with men?	0.48	0.71
4.How often have you been hit or beaten up because you have sex with men?	0.21	0.80
5.How often have you had to pretend that you are not homosexual in order to be accepted?	0.63	0.06
6.How often has your family not accepted you because you have sex with men?	0.50	0.70
7.How often have you lost your friends because you have sex with men?	0.50	0.71
8.How often have you been kicked out of school for being homosexual?	0.07	0.84
9.How often have you lost a place to live because you have sex with men?	0.22	0.84
10.How often have you lost a job or career opportunity because you have sex with men?	0.19	0.84
11.How often have you experienced police harassment because you have sex with men?	0.25	0.76

**Fig. 2:**
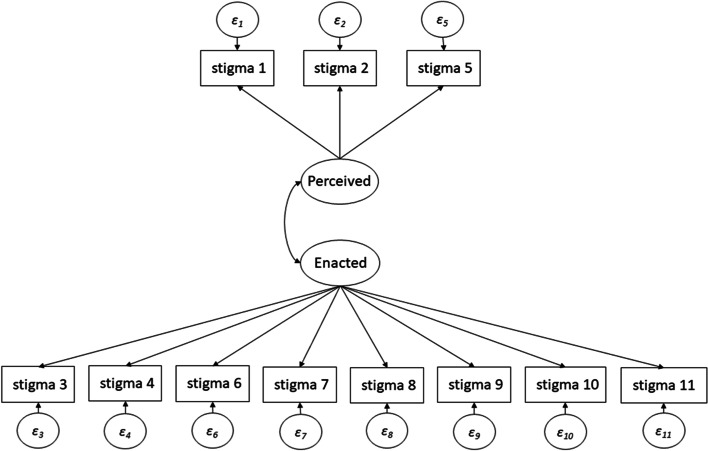
Hypothesized Neilands sexual stigma scale model

Three items (item 3, “How often have you been made fun of or called names because you have sex with men,” item 6, “How often has your family not accepted you because you have sex with men?” and item 7 “How often have you lost your friends because you have sex with men?”) had loadings of more than 0.40 on both factors. The difference between loadings was 0.20 or more for all three items (item 3 difference = 0.23, item 6 difference = 0.20, item 7 difference = 0.21, Table 3). All three loaded more strongly on the enacted stigma factor. The final hypothesized model is depicted in Fig. 2.

### Confirmatory factor analysis

A two-factor CFA was conducted using the random sample of 436 participants not included in the EFA. Items cross-loading on both factors were included on the enacted stigma factor, where they loaded most strongly. Fit statistics for the initial two-factor model indicated an acceptable fit for all measures except the chi-squared, which was significant at *p* < 0.01, indicating a poor fit.

A one-factor CFA conducted on the same sample indicated a similar fit to the two-factor model, while site-specific two-factor CFAs had similar fit to that of the two-factor CFA using the randomly selected CFA sample. Table 4 presents fit statistics for these models. Cronbach’s alpha for the CFA sample was 0.58 for the perceived factor, indicating questionable internal consistency, and 0.88 for the enacted factor, indicating good internal consistency.

**Table 4:** Model fit statistics for the Neilands sexual stigma scale

		CFA Sample		Nairobi	Coastal Kenya
	One factor 11-item model	Two factor 11-item model	Two factor 8-item model*	Two factor 11-item model	Two factor 11-item model
Chi-squared (df) *p*-value	240.9 (44) *p* < 0.01	209.7 (43) *p* < 0.01	26.5 (17) *p* = 0.07	246.0 (43) *p* < 0.01	158.4 (43) *p* < 0.01
Root mean square error of approximation (RMSEA)	0.10	0.09	0.04	0.10	0.09
Comparative-fit index (CFI)	0.95	0.96	0.996	0.97	0.96
Standardized root mean squared residual (SRMR)	0.07	0.06	0.03	0.06	0.07

^*^ Items and item correlations show in Fig. 3

### Model re-specification

**Fig. 3:**
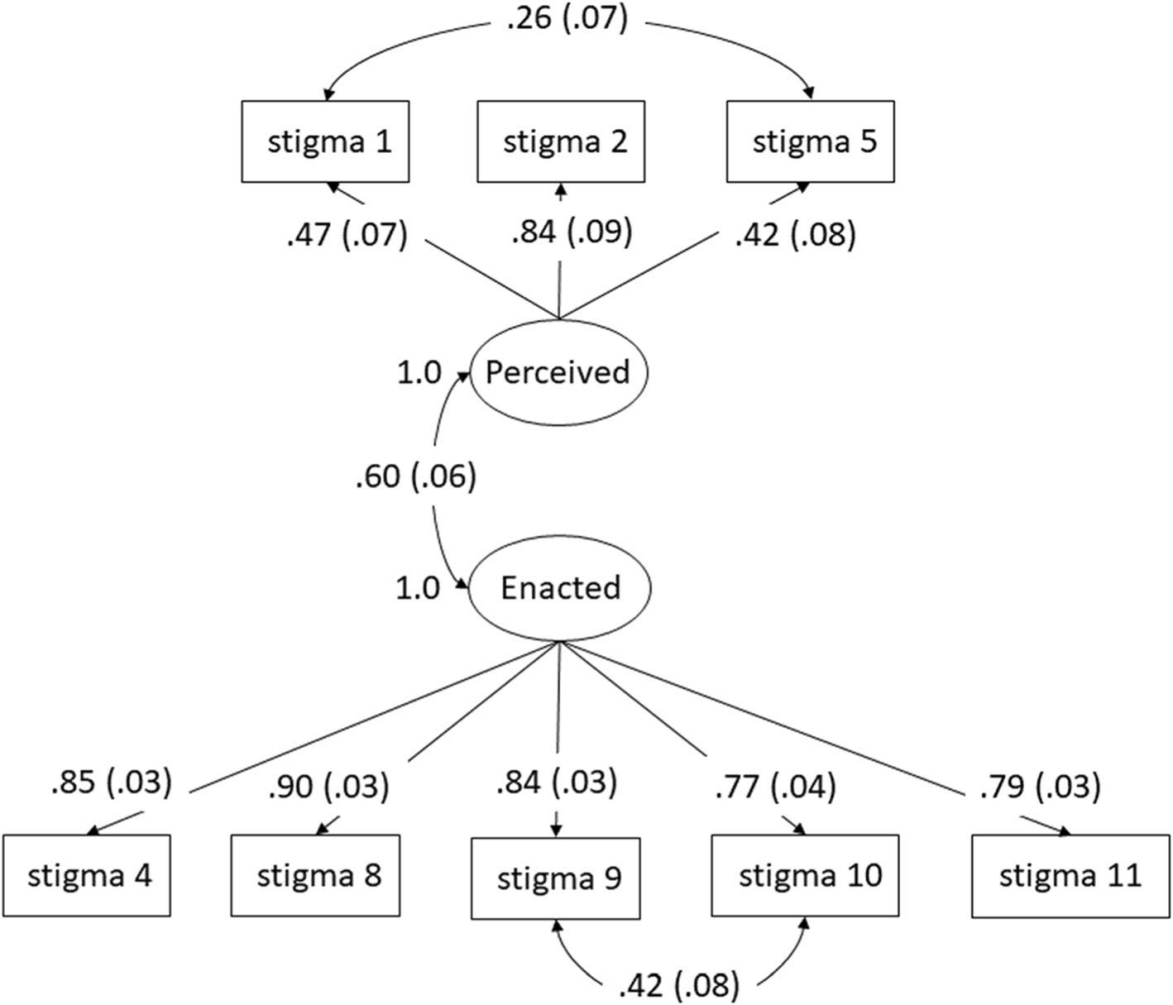
Final 8-item sexual stigma scale model with standardized loadings

To improve model fit, we considered cross-loading and modification indices indicating high residual correlation to identify changes in the model. Item 3 cross-loaded on both factors, so was removed and the fit statistics re-run. This change produced a chi-squared of 136.7 (df 32), *p* < 0.01. The other two cross-loaded items (item 6 and item 7), which also had the highest modification indices, were subsequently removed to further improve model fit. After adding error correlation, the final 8- item model had a chi-squared value of 26.5 with 17 df, *p* = 0.07 and good fit on the other indices (Table 4, Fig. 3).

### Construct validity associations

Eleven participants who did not answer one or more items on the PHQ-9, AUDIT, or DAST-10 were excluded from the construct validity analysis, leaving 860 participants. For the final 8-item model, perceived stigma was associated with PHQ-9 score (beta = 0.34, 95% CI 0.24, 0.45), alcohol use (beta = 0.14, 95% CI 0.03, 0.25), and other substance use (beta = 0.19, 95% CI 0.07, 0.31), while enacted stigma was associated with AUDIT score (beta = 0.17, 95% CI 0.06, 0.27) (Table 5). Using the initial 11-item model, perceived stigma was associated with PHQ-9 score (beta = 0.35, 95% CI 0.20, 0.51), and enacted stigma was associated with AUDIT score (beta = 0.21, 95% CI 0.07, 0.33).

**Table 5:** Association of the perceived and enacted factors of the Neilands sexual stigma scale with depressive symptoms (PHQ-9), alcohol use (AUDIT), and other substance use (DAST-10), comparing the final 8-item model and initial 11-item model, *n* = 860

β (95% CI)	PHQ-9	AUDIT	DAST-10
Final 8-item model			
Perceived stigma	0.34 (0.24, 0.45)	0.14 (0.03, 0.25)	0.19 (0.07, 0.31)
Enacted stigma	0.10 (-0.01, 0.20)	0.17 (0.06, 0.27)	0.10 (-0.01, 0.21)
Initial 11-item model			
Perceived stigma	0.35 (0.20, 0.51)	0.09 (-0.07, 0.24)	0.17 (-0.01, 0.34)
Enacted stigma	0.07 (-0.08, 0.22)	0.21 (0.07, 0.33)	0.11 (-0.05, 0.26)

## Discussion

These results provide validation of an adapted version of the Neilands sexual stigma scale with perceived and enacted stigma measures tailored for use with Kenyan GBMSM. Reliability, or internal consistency, of the summed sexual stigma scale was good both overall and at each research site. Exploratory analysis revealed a two-factor structure, while confirmatory analysis showed that using all items resulted in suboptimal model fit. Post-hoc analysis, in which three enacted stigma items which cross-loaded on both factors were removed and correlated errors added, produced a better fitting model. Construct validity analysis demonstrated associations between perceived stigma and PHQ-9, AUDIT, and DAST-10 scores, and between enacted stigma and AUDIT score, further supporting the validity of the scale.

Perceived stigma relates to one’s beliefs about others’ opinions. Items representing the perceived stigma factor asked about opinions held by others (i.e., item 1, “How often have you heard that homosexuals are not normal?”), one’s actions based on those opinions (i.e., item 5, “How often have you had to pretend that you are not homosexual in order to be accepted?”), and other’s reactions based on their opinions, (i.e., item 2, “How often have you felt that you hurt and embarrassed your family because you have sex with men?”). The perceived stigma factor, which contained only three items, had poor reliability. More items addressing community opinions about GBMSM and reactions to those opinions should be tested in order to increase the comprehensiveness of this factor. For example, Logie and Earnshaw [37] added two items to their perceived stigma scale for sexual minority women: “How often have you heard that lesbian, bisexual and queer women grow old alone?” and “How often have you felt you had to stop associating with your family because you are lesbian, queer or bisexual?” Adding items such as these to the Neilands sexual stigma scale may better represent the concept of perceived stigma among GBMSM as well, making this factor more stable and distinct from the enacted stigma factor.

Enacted stigma relates to actions of others. Items loading on this factor measured experiences of violence, discrimination, and loss of opportunity. Individuals endorsing these items may have a greater need for assistance and support. In coastal Kenya, 67% of GBMSM had experienced emotional, physical, or sexual abuse in the previous year, demonstrating the vulnerability of this population [21]. Disclosure of sexual orientation is particularly fraught and has been associated with blackmail and abuse in other rights-constrained African settings [11, 38]. While structural- and community-level interventions are needed to address stigma and discrimination [39, 40], individual-level interventions, such as learning about resilience from positive outliers, can help support GBMSM who have experienced enacted stigma [41].

In our analysis, three items loaded significantly on both the perceived and the enacted factors. These items, (3, 6, and 7) all represent perceptions of other’s actions and were dropped from our final model. Similarly, item 3 cross-loaded in Neilands’ initial factor analysis and was subsequently dropped [18]. Our changes to the model, including the addition of correlated error, resulted in a chi-squared test statistic indicating a good model fit. Using the chi-squared statistic to assess model fit has limitations, as the statistic is sensitive to sample size, and larger samples decrease the chi-squared p-value [35]. However, since poor model fit likely indicates misspecification, and misspecified models can be unpredictably biased, the conservative option is to adjust the model to improve chi-square fit [35].

Our analysis of construct validity looked at associations between each factor and both mental health and substance use outcomes, comparing the final 8-item model and the initial 11-item model. The enacted sexual stigma factor was associated with only alcohol use in both the initial and final model. This supports the conclusion that the enacted stigma factor is a good representation of its construct. However, the perceived stigma factor was less consistent. There was an association between perceived stigma and depressive symptoms using both models. For alcohol use and other substance use, the association point estimates (betas) were similar, but the associations were statistically significant in the final model only. This indicates that the perceived stigma factor is less stable, a conclusion also reached by Tucker et al., who found insufficient reliability in the perceived stigma scale [42].

This study had several limitations. First, while perceived and enacted sexual stigma were both measured, internalized sexual stigma was not. Internalized sexual stigma, or internalized homophobia, is a personalized endorsement of negative beliefs against GBMSM and has been associated with numerous negative mental health outcomes [43]. Not including this factor in the analysis may have overestimated the effects of the perceived and enacted stigma factors. Second, many participants experience additional stigmas from sources not measured here, such as HIV status or sex work, and stigmatizing experiences attributed by the participant to their other identities may underestimate the effects of sexual stigma [14]. Third, we did not measure “outness” in this study, and GBMSM who are more out to society may experience more enacted stigma compared to those who are not yet out. It is possible that outness could be considered as a potential confounder or modifier of the association between sexual stigma and health outcomes [44]. Fourth, participants were volunteers who were engaged in HIV care and prevention services targeting GBMSM, including those who sell sex, and are not representative of all GBMSM in Kenya. Finally, cross-sectional data were used, so the causal directionality of associations cannot be determined.

Despite these limitations, our study has several strengths. First, this is one of the few studies in sub-Saharan Africa to look at two types of sexual stigma, both perceived and enacted. Second, the study had a relatively large sample size that included a diverse population of GBMSM from Nairobi, the capital and largest city of Kenya, and from smaller communities in coastal Kenya north of Mombasa, Kenya’s second largest city. Third, collaboration fostered by the Kenya MSM Health Research Consortium (msmhealthresearch.org) allowed inclusion of the same item wording in cohort studies in two locations, allowing for a more comprehensive view of sexual stigma among GBMSM in Kenya.

## Conclusions

Sexual stigma is commonly reported among GBMSM in Kenya, and can result in a substantial burden of physical, mental, and emotional abuse. Our exploratory factor analysis of the Neilands sexual stigma scale as modified for use in Kenya produced two factors: perceived and enacted sexual stigma. Confirmatory factor analysis corroborated two distinct factors, and three cross-loading items were dropped from the enacted factor to improve fit. Enacted and perceived stigma were associated with important measures of health: perceived stigma with depressive symptoms, alcohol use, and other substance use and enacted stigma with alcohol use. The enacted stigma factor showed more stability than the perceived factor when looking at associations using the initial and final model. Overall, the Neilands sexual stigma scale is a valid measure of sexual stigma among Kenyan GBMSM.

## Data Availability

Data and data dictionary is available through public access on-line repository at https://dataverse.harvard.edu/dataverse/kwtrp.

## Abbreviations

ACASI: Audio computer-assisted self-interview
AUDIT: Alcohol Use Disorder Identification Test
CFA: Confirmatory factor analysis
CFI: Comparative-fit index
DAST: Drug Abuse Screening Test 10
EFA: Exploratory factor analysis
GBMSM: Gay, bisexual, and other men who have sex with men
HIV: Human immunodeficiency virus
HOYMAS: Health Options for Young Men on HIV/AIDS and STIs
KEMRI-WTRP: Kenya Medical Research Institute-Wellcome Trust Research Programme
MSM: Men who have sex with men
NASCOP: National AIDS and STI Control Programme (Kenya)
PHDA: Partners for Health and Development in Africa
PHQ-9: Patient Health Questionnaire 9
RMSEA: Root mean square error of approximation
SRMR: Standardized root mean squared residual
UNAIDS: Joint United Nations Progamme on HIV/AIDS
WHO: World Health Organization
WLSMV: Weighted least squares with adjustment for means and variances estimation for categorical variables
